# Decoupling of Global Brain Activity and Cerebrospinal Fluid Flow Is Linked to Alzheimer's Pathology

**DOI:** 10.1093/geroni/igab046.2413

**Published:** 2021-12-17

**Authors:** Feng Han, Jing Chen, Aaron Belkin-Rosen, Yameng Gu, Liying Luo, Orfeu Buxton, Xiao Liu

**Affiliations:** 1 the Pennsylvania State University, State College, Pennsylvania, United States; 2 The Pennsylvania State University, State College, Pennsylvania, United States; 3 The Pennsylvania State University, University Park, Pennsylvania, United States; 4 The Pennsylvania State University, The Pennsylvania State University, Pennsylvania, United States

## Abstract

Alzheimer’s disease (AD) is the most common cause of dementia in the old adult population. AD pathogenesis has been linked to the aggregation of toxic proteins, e.g., amyloid-β and tau. The glymphatic system may play an important role in clearing out these proteins via cerebrospinal fluid (CSF) flows through perivascular and interstitial spaces. Recent studies have suggested low-frequency (<0.1 Hz), sleep-dependent global blood-oxygenation-dependent-level (gBOLD; global resting-state functional MRI signal) during resting state is coupled with CSF movements, suggesting their potential link to glymphatic function. Here, we directly investigated whether the coupling between the gBOLD and CSF signals is related to AD-related pathology. By analyzing neuroimaging, neurobiological, and neuropsychological data from 118 human subjects (58-90 years of age; AD, early-stage AD, and control subjects included) collected in the Alzheimer's Disease Neuroimaging Initiative project, we found a strong coupling between the gBOLD and CSF signals. More importantly, the strength of this gBOLD-CSF coupling was significantly correlated with cortical amyloid-β level (p = 0.019), cognitive decline in the subsequent two years (p = 0.013), disease severity (p = 0.035), and several AD-related risk factors, including aging (p = 0.011), and gender (p = 0.026). These findings provide initial evidence for the critical role of resting-state low-frequency (<0.1 Hz) neural/physiological dynamics in AD pathology. They also suggest that the gBOLD-CSF coupling may serve as a non-invasive imaging marker for gauging the glymphatic function.

